# California Prescribed Fire Monitoring Program: dataset 2019-2024

**DOI:** 10.1038/s41597-025-06509-0

**Published:** 2026-02-10

**Authors:** Rut Domènech, Anna Maria Naimeh, Tessa Putz, Ashley R. Grupenhoff, Melanie Schlueter, Ryan M. Boynton, John Williams, David B. Sapsis, Joseph Restaino, Nadia Tase, Becky L. Estes, Hugh D. Safford

**Affiliations:** 1 Department of Environmental Science and Policy, University of California, Davis, California USA; 2 Department of Natural Resources and Environmental Science, Cal Poly San Luis Obispo, San Luis Obispo, California USA; 3 Fire and Resource Assessment Program, California Department of Forestry and Fire Protection, Sacramento, California USA; 4 USDA Forest Service, Eldorado National Forest, Placerville, CA USA

**Keywords:** Fire ecology, Environmental impact

## Abstract

The California Prescribed Fire Monitoring Program dataset (2019–2024) provides comprehensive ecological monitoring data from prescribed fire treatments across California’s diverse forest ecosystems. This dataset encompasses forest structure and cover, fuel loads, and post-fire recovery metrics, collected using a standardized protocol, from over 36 disparate sites (114 burn units, 972 plots, and 1,838 total surveys). Data collected during pre-fire, immediate, and multi-year post-fire sampling episodes allow for robust analysis of prescribed fire effects across variable environmental conditions. The monitoring framework captures key ecological indicators, including tree mortality, fuel consumption, understory vegetation response, species composition, and regeneration. This dataset can address critical knowledge gaps regarding prescribed fire effectiveness for ecological restoration, hazardous fuel reduction, and ecosystem resilience objectives. These data can support evidence-based fire management decisions, validate fire effects models, and establish baseline reference conditions for future prescribed fire implementation throughout California’s fire-prone landscapes.

## Background & Summary

California’s ecosystems evolved with fire as a natural ecological process^1–4^. However, 150 years of intense fire management practices and policies have resulted in major ecosystem changes. In much of the California forested land base, loss of large fire-tolerant trees, stand densification by fire-intolerant species, and fuel accumulations are contributing to increasingly severe wildfires that threaten ecological integrity, human lives, and infrastructure^1,5,6^. Prescribed fire has emerged as a critical tool for restoring natural fire regimes, reducing hazardous fuels, and improving ecosystem resilience to climate change across California’s diverse landscapes^7,8^.

Despite widespread recognition of prescribed fire’s value, comprehensive datasets documenting its ecological effects and fuel hazard reduction benefits across California’s varied ecosystems remain scarce. While individual prescribed fire projects sometimes include monitoring, these efforts typically follow project-specific protocols with limited spatial and temporal scope, hampering broader synthesis and application^9^. The California Prescribed Fire Monitoring Program (CPFMP) was established by the California Department of Forestry and Fire Protection (CAL FIRE) in 2019 to address this critical knowledge gap through standardized, long-term ecological monitoring across the state’s diverse prescribed fire users and practices^10–12^. Development of field data collection protocols, field data collection, and database management for the 2019–2024 period was completed in partnership with the University of California – Davis (UC Davis).

This dataset represents a comprehensive, standardized monitoring of prescribed fire treatments spanning California’s major forest and woodland types, including Sierra Nevada mixed conifer, Sierra Nevada eastside pine, coastal redwoods, oak woodlands, and montane hardwood. Data were collected before treatment, immediately after treatment, and at several-year intervals post-fire. The monitoring framework captures multiple ecological indicators, including vegetation structure and cover, species composition, fuel loads, and regeneration.

The resulting dataset provides an unparalleled opportunity to evaluate prescribed fire effectiveness across diverse ecological contexts, quantify variability in fire effects, and refine predictive models for fire managers. As California and other western states seek to dramatically increase the pace and scale of prescribed fire implementation, these data will support evidence-based decision-making, refine fire effects and fire behavior models, and help establish ecologically appropriate prescribed fire regimes for fire-adapted ecosystems throughout the region.

## Methods

### Site description

**Fig. 1:**
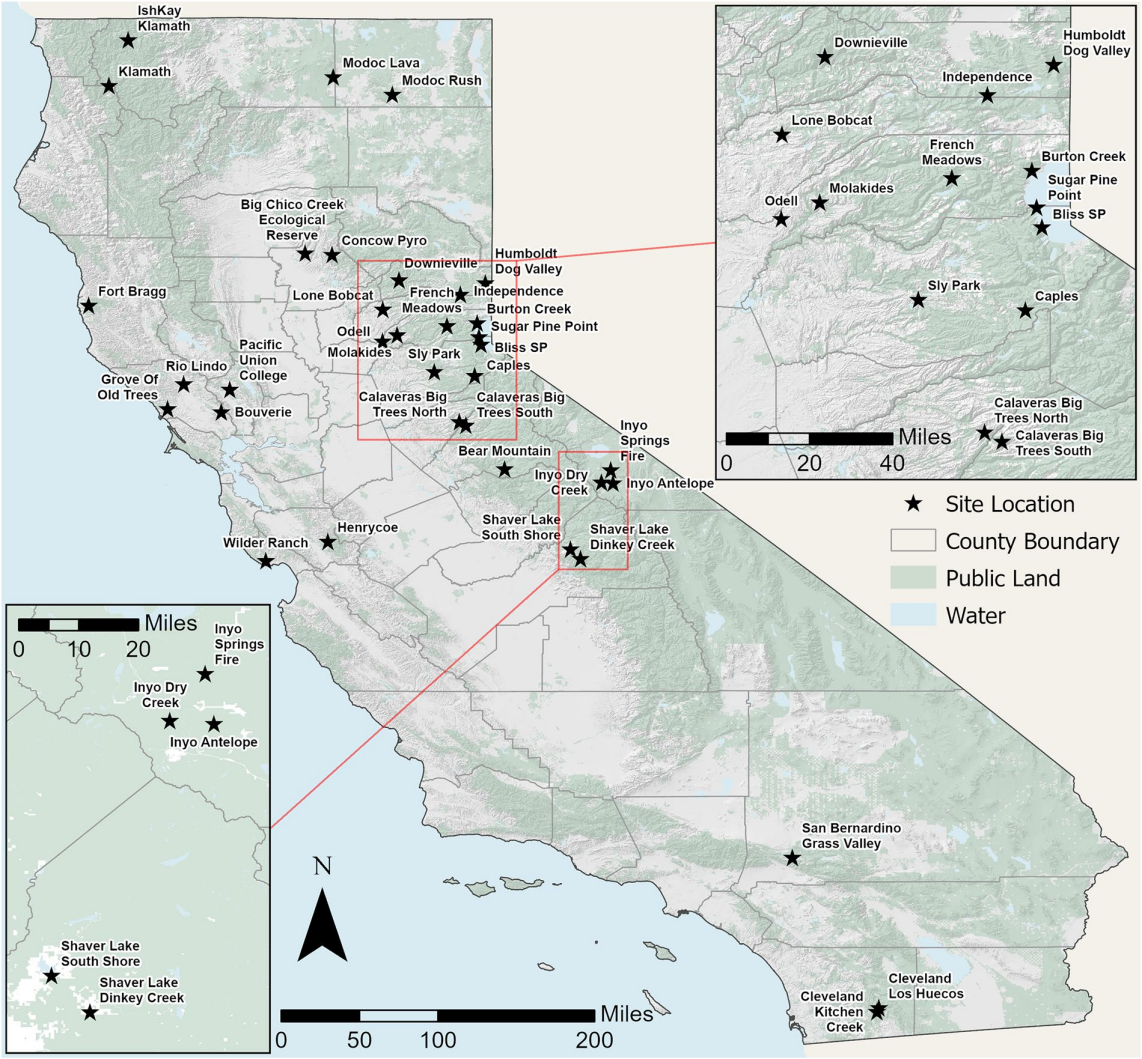
Study site locations. Sites: Big Chico Creek Ecological Reserve, Bliss SP, Bouverie, Burton Creek, Calaveras Big Trees, Caples, Cleveland Kitchen Creek, Cleveland Los Huecos, Concow Pyro, Downie Ville, French meadows, Fort Bragg, Grove Of Old Trees, Henrycoe, Humboldt Dog Valley, Independence, Inyo Antelope, Inyo Dry Creek, Inyo Springs Fire, Ish Kay Klamath, Klamath, Lone Bobcat, Modoc Lava, Modoc Rush, Molakides, Odell, Pacific Union College, Rio Lindo, San Bernardino Grass Valley, Shaver Lake, Sly Park, Sugar Pine Point, Wilder Ranch.

Sites were strategically selected across California to capture the geographic, ecological, and jurisdictional diversity of prescribed fire applications statewide (Fig. 1). Our monitoring network comprises 36 sites chosen based on four criteria: (1) planned prescribed burns with sufficient lead time for pre-treatment sampling; (2) accessibility for repeat sampling; (3) representation across major forest ecosystem types; and (4) distribution across ownership types (federal, state, private).

General climatic patterns at the study sites are broadly similar (although temperature profiles vary due to different elevations and proximity to the coast), with most of them found within the North American Mediterranean Climate Zone. All sites are characterized by moist and cool winters and dry and warm or hot summers (Köppen climate types Csb and Csa); altitudes range from 68 m to 2,492 m (Table 1).

**Table 1:** Description of the study sites.

Code	Site	Ownership	Elevation (m)	Mean annual precip (mm)	Minimum temp of coldest month (°C)	Maximum temp of warmest month (°C)	Forest type	Plot Count
1	Bear Mountain	Stanislaus National Forest	1,450	1,007	−1	29	Sierran Mixed Conifer	27
2	Big Chico Creek Ecological Reserve	California State University Chico / Big Chico Creek Ecological Reserve	412	1,166	4	34	Montane Hardwood	11
3	Bliss SP	CA State Parks	1,983	974	−6	26	Sierran Mixed Conifer	15
4	Bouverie	Fire Forward / Audubon Canyon Ranch	157	883	4	29	Coastal Oak Woodland	12
5	Burton Creek	CA State Parks	2,029	1,025	−6	25	Sierran Mixed Conifer	47
6	Calaveras Big Trees North	CA State Parks	1,469	1,243	−1	29	Sierran Mixed Conifer	34
7	Calaveras Big Trees South	CA State Parks	1,535	1,237	−1	29	Sierran Mixed Conifer	58
8	Caples	Eldorado National Forest	1,979	1,233	−4	26	Sierran Mixed Conifer	108
9	Cleveland Kitchen Creek	Cleveland National Forest	1,720	645	−1	29	Montane Hardwood Conifer	43
10	Cleveland Los Huecos	Cleveland National Forest	1,753	643	−1	29	Montane Hardwood Conifer	25
11	Concow Pyro	Plumas National Forest / Concow Pyrodiversity Project	1,019	1,718	2	31	Sierran Mixed Conifer	17
12	Downieville	Tahoe National Forest / Red Ant Fuels Reduction Project	1,568	1,787	−2	28	Sierran Mixed Conifer	31
13	Fort Bragg	Jackson Demonstration State Forest / Redwood Resiliency Project	226	1,179	4	24	Redwood	30
14	French Meadows	Tahoe National Forest/ The Nature Conservancy	1,918	1,642	−3	26	Sierran Mixed Conifer	77
15	Grove Of Old Trees	Fire Forward / LandPaths / Point Blue	350	1,479	5	27	Redwood	15
16	Henrycoe	CA State Parks	692	582	5	30	Coastal Oak Woodland	14
17	Humboldt Dog Valley	Humboldt-Toiyabe National Forest	1,883	587	−6	29	Eastside Pine	16
18	Independence	The Nature Conservancy	2,176	1,212	−7	25	Sierran Mixed Conifer	41
19	Inyo Antelope	Inyo National Forest	2,306	445	−9	27	Eastside Pine	19
20	Inyo Dry Creek	Inyo National Forest	2,351	500	−9	26	Eastside Pine	26
21	Inyo Springs Fire	Inyo National Forest	2,560	554	−9	25	Eastside Pine	51
22	IshKay Klamath	Mid-Klamath Watershed Council / Karuk Tribe	374	1,274	0	34	Douglas Fir	13
23	Klamath	Mid-Klamath Watershed Council / Karuk Tribe	314	1,696	2	34	Douglas Fir	18
24	Lone Bobcat	Private	937	1,485	1	32	Douglas Fir	7
25	Modoc Lava	Modoc National Forest	1,412	681	−5	29	Sierran Mixed Conifer	41
26	Modoc Rush	Modoc National Forest	1,464	460	−5	29	Sierran Mixed Conifer	12
27	Molakides	Private	911	1,367	2	32	Montane Hardwood	5
28	Odell	Private	555	1,038	3	33	Montane Hardwood	5
29	Pacific Union College	Pacific Union College	546	1,003	4	31	Coastal Oak Woodland	20
30	Rio Lindo	Fire Forward / Rio Lindo Adventist Academy	60	1,067	4	30	Coastal Oak Woodland	9
31	San Bernadino Grass Valley	San Bernardino National Forest	1,669	645	−1	29	Montane Hardwood Conifer	10
32	Shaver Lake Dinkey Creek	Southern California Edison (SCE)	1,760	966	−3	27	Sierran Mixed Conifer	45
33	Shaver Lake South Shore	Southern California Edison (SCE)	1,740	960	−2	28	Sierran Mixed Conifer	26
34	Sly Park	Sierra Pacific Industries, El Dorado Irrigation District	1,168	1,256	1	31	Sierran Mixed Conifer	22
35	Sugar Pine Point	CA State Parks	1,905	968	−6	26	Sierran Mixed Conifer	2
36	Wilder Ranch	CA State Parks	223	1,047	5	25	Redwood	20

Bioclimatic Variables calculated using BCM (Basin Characterization Model) from 1991–2020^18^.

The dataset includes conifer-dominated forest types such as California mixed conifer, eastside Jeffrey pine (found east of the Sierra Nevada crest), as well as moister redwood and Douglas-fir forests. Broadleaf-dominated forest types represented include California montane hardwood and coastal oak woodland (Table 1).

Major tree species in the dataset include ponderosa pine (*Pinus ponderosa* Laws), Jeffrey pine (*P. jeffreyi* Grev. & Balf.), sugar pine (*Pinus lambertiana* Dougl.), white fir (*Abies concolor* Gord. & Glend), red fir (*A. magnifica* A. Murray bis), redwood (*Sequoia* s*empervirens* (Lamb. ex D. Don) Endl.), incense-cedar (*Calocedrus decurrens* (Torr.) Floren.), coast live oak (*Quecus agrifolia* Née), interior live oak (*Quercus wislizeni* A. DC.), California black oak (*Quercus kelloggii* Newb.), and California bay (*Umbellularia californica* (Hook. & Arn.) Nutt.).

### Prescribed fire treatments

**Fig. 2:**
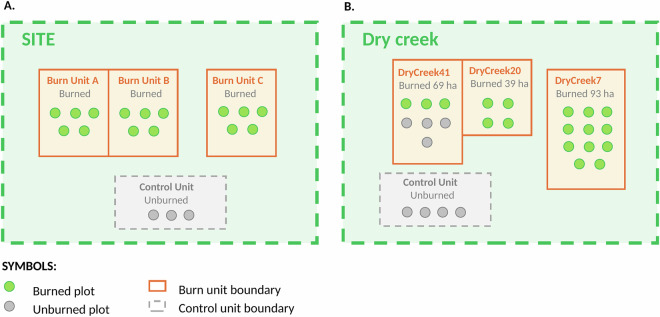
Hierarchical spatial sampling design. (**A**) Schematic showing nested design with sites containing burn units, which contain monitoring plots (0.04 ha, 11.4 m radius). Burn units may be adjacent or separated. Not all burn units burned at the same time, some serve as unburned controls. Green circles indicate burned plots; gray circles indicate control plots. (**B**) Dry Creek site example with three burned units (DryCreek41, 69 ha; DryCreek20, 39 ha; DryCreek7, 93 ha) and one control unit. Design allows multi-scale analysis across 36 sites (114 burn units, 972 plots).

The study encompassed 36 sites containing a total of 114 burn units (1 to 11 burn units per site), and 972 plots (1 to 108 plots per burn unit). Burn units corresponded to operational units for fire management and were placed adjacent or separated depending on operational constraints and management objectives (Fig. 2). Between 2019 and 2024, prescribed fires were conducted in 57 of these burn units, covering 381 plots. The primary objective of these prescribed fires was fire risk reduction. Additionally, 11 burn units (153 plots) experienced wildfires during this period (2019–2024), including the Springs Fire, which was a lightning ignition managed for resource benefit, and the Caples Fire that initiated as a prescribed fire and was later converted to a wildfire following an escape. Prior to prescribed burning, some burn units received other fuel treatments, including thinning and pile burning, as part of integrated fuel management approaches (Table 2).

**Table 2:** Description of the burn units.

Code	Site	Burn Unit	Number of Plots	Type of treatment	Surface (ha)	Burn Date
1	Bear Mountain	BM1	6(6)	RxF	30.1	September 25, 2019
		BM2	6(6)	RxF	68.4	September 26, 2019
		BM3	3(3)	RxF	40.3	September 29, 2019
		BM4	12(12)	RxF	113.5	September 30, 2019
2	Big Chico Creek Ecological Reserve	BCCER_undetermined	11(11)	W	28.7	August 8, 2024*
3	Bliss SP	bliss_central	4(0)	na	13.2	Not Burned
		bliss_north	6(0)	na	13.0	Not Burned
		bliss_south	5(0)	na	16.3	Not Burned
4	Bouverie	cougar	5(0)	na	2.2	Not Burned
		fins	5(0)	na	2.8	Not Burned
		gilman	2(2)	RxF	0.9	December 11, 2021
5	Burton Creek	B1	5(5)	RxF	7.7	November 13, 2019
		B2	5(5)	RxF	7.9	September 27, 2023
		B3	5(5)	RxF	8.1	October 16, 2023
		B4	8(8)	RxF	8.5	October 17, 2023
		BUc	6(0)	TP	10.8	Not Burned
		BUd	5(5)	RxF	7.6	October 19, 2015
		control	13(0)	na	na	Not Burned
6	Calaveras Big Trees State Park - North Grove	6	2(2)	RxF	2.4	May 16, 2022
		7	12(12)	RxF	25.3	October 23–26, 2022
		8	20(20)	RxF	32.6	May 16, 2022
7	Calaveras Big Trees State Park - South Grove	south_grove	58(58)	RxF	527.4	October 29 – November 1, 2024*
8	Caples^1^	Cap0	108(102)	W	1,390.1	September 30-October 25, 2019
9	Cleveland Kitchen Creek	40	4(4)	TPB	7.5	December 20, 2023
		41	8(8)	TPB	13.4	November 13, 2023
		42a	12(11)	TPB	25.4	November 14, 2023
		42b	5(5)	TPB	11.1	November 28, 2024
		47	14(14)	TPB	21.9	January 31, 2024
10	Cleveland Los Huecos	57	5(0)	na	10.8	Not burned
		58	12(11)	TPB	63.7	December 2023 to May 2024
		59	8(5)	TPB	37.2	December 2023 to May 2024
11	Concow Pyro	1a	2(0)	na	239.5	Not burned
		2a	8(3)	RxF	142.5	December 11, 2024*
		8a	7(0)	na	70.2	Not burned
12	Downieville	RedAnt	21(0)	na	121.8	Not burned
		control	10(0)	na	na	Not burned
13	Fort Bragg	HEN	6(0)	na	38.0	Not burned
		KAY	6(0)	na	16.0	Not burned
		TRE	4(0)	na	14.2	Not burned
		UQL	4(0)	na	12.9	Not burned
		WIL	5(0)	na	26.3	Not burned
		ZIE	5(0)	na	24.4	Not burned
14	French meadows	10	15(0)	na	40.5	Not burned
		17 A	15(0)	na	1,392.8	Not burned
		19 A	11(11)	RxF	65.5	May 16, 2021
		19B	4(0)	RxF	42.9	Not burned
		19 C	6(0)	RxF	17.9	Not burned
		19D	13(0)	na	25.7	Not burned
		19E	13(0)	na	60.1	Not burned
15	Grove Of Old Trees	Road	2(0)	na	na	Not burned
		Zone1	4(4)	RxF	1.1	November 20, 2022
		Zone2	4(4)	RxF	1.3	November 20, 2022
		Zone3	5(5)	RxF	2.8	May 27, 2025*
16	Henrycoe	HC0	14(8)	RxF	13.9	November 14, 2023
17	Humboldt Dog Valley	DV7	16(16)	RxF	65.7	May 16, 2023
18	Independence	A	4(0)	na	220.4	Not burned
		IND0	37(0)	na	197.1	Not burned
19	Inyo - Antelope	antelope11b	4(2)	RxF	64.2	June 23–25, 2023
		antelope2	15(15)	RxF	234.2	June 23–25, 2023
20	Inyo - DryCreek	control	4(0)	na	na	Not burned
		drycreek20	4(4)	RxF	39.1	November 15–18, 2021
		drycreek41	7(3)	RxF	69.0	November 15–18, 2021
		drycreek7	11(11)	na	93.1	October 23–24, 2018
21	Inyo - Springs Fire	alpha	9(9)	W	2,676.8	July 26 to August 20, 2019
		bravo	3(3)	W		July 26 to August 20, 2019
		charlie	4(4)	W		July 26 to August 20, 2019
		controlA	5(0)	na		Not burned
		controlB	6(0)	na		Not burned
		delta	3(3)	W		July 26 to August 20, 2019
		echo	1(1)	W		July 26 to August 20, 2019
		foxtrot	5(5)	W		July 26 to August 20, 2019
		golf	5(5)	W		July 26 to August 20, 2019
		hotel	4(4)	W		July 26 to August 20, 2019
		springs_unknown	6(6)	W		July 26 to August 20, 2019
22	IshKay Klamath	Ish_Kaysh40	13(0)	na	66.4	Not burned
23	Klamath	KLA0	18(2)	RxF	140.0	2021
24	Lone Bobcat	lbw_A	2(0)	na	6.9	Not burned
		lbw_B	2(0)	na		Not burned
		lbw_C	3(1)	RxF		April 7th, 2021
25	Modoc Lava	10	6(6)	RxF	21.2	May 10, 2021
		211	4(0)	na	24.7	Not burned
		212	2(0)	na	12.0	Not burned
		3000	2(2)	RxF	5.6	May 11, 2021
		5	2(2)	RxF	12.6	May 10, 2021
		910	6(6)	RxF	23.2	May 11, 2021
		control	8(0)	na	na	Not burned
		controlB	5(0)	na	na	Not burned
		mast1	4(0)	na	17.1	Not burned
		mast2	2(0)	na	13.2	Not burned
26	Modoc Rush	Rush102	8(8)	RxF	30.8	September 27, 2023
		Rush120	4(4)	RxF	23.6	September 27, 2023
27	Molakides	2022-01	5(0)	na	12.5	Not burned
28	Odell	ODELL0	5(3)	RxF	6.8	February 8, 2022
29	Pacific Union College	1	1(1)	RxF	1.8	May 15, 2025*
		4	9(0)	na	1.9	Not burned
		6a	4(4)	RxF	9.1	May 1, 2024
		9	6(0)	na	14.3	Not burned
30	Rio Lindo	NorthA	6(6)	RxF	3.0	June 17, 2022
		NorthB	3(3)	RxF	1.6	June 17, 2022
31	San Bernardino Grass Valley	eldorado	10(0)	na	45.1	Not burned
32	Shaver Lake Dinkey Creek	Dinkey_C	10(2)	PB	11.6	December, 2023
		Dinkey_D	16(5)	PB	20.4	December, 2023
		Dinkey_E	6(1)	PB	na	December, 2023
		Dinkey_F	13(9)	PB	24.4	December, 2023
33	Shaver Lake SouthShore	A	12(5/4)	RxF	69.4	May 19, 2022/October, 2023
		B	10(0)	na	51.1	Not burned
		C	4(0)	na	4.3	Not burned
34	Sly park	hcp-east	7(0)	na	9.0	Not burned
		hcp-west	4(0)	na	114.3	Not burned
		spiA	6(6)	RxF	17.2	February 11 and 20, 2020
		spiB	5(5)	RxF	16.1	February 11, 2020
35	Sugar Pine Point	SP0_1	1(1)	RxF	1.5	October 4, 2022
		SP0_2	1(1)	RxF	3.1	October 17, 2022
36	Wilder Ranch	RNSP0	20(12)	RxF	152.4	November 25–27, 2022

Number of Plots: In each burn unit, not all plots burned; in brackets, the plots that burned are detailed. If in the same burn unit there was to different burn dates that affected different plots, then they are separated with “/”. Type of treatment: RxF, Prescribed fire; W, Wildfire; TPB, Thin, Pile and Burn; PB, pile burning, TP, Thin and pile; T, Thin; na, not applicable. Burn date: * indicates that no post-treatment data is included in this dataset.

^1^The Caples project started on Sep 30, 2019 and proceeded until Oct 10, 2019 - at which point it was declared a wildfire. About 437.1 ha were burned as prescribed fire, with an additional 953.0 ha burned after the fire was declared a wildfire. Pre-fire data (2013–2018) was collected by USDA FS crews. Pre-fire data does not have tree height The USDA FS has continued to collect data within the Caples project following Caldor Fire of which some Caples plots were reburned. Please contact Becky Estes, becky.estes@usda.gov for interest in the Caples project data.

Prescribed fires occurred year-round, depending on the weather, fuel condition, and available personnel. The study sites were managed by various landowners, including the USDA Forest Service, California State Parks, and The Nature Conservancy, among others. Burned units varied considerably in size, ranging from 2.3 to 234.2 ha for prescribed fires, while the Springs Fire covered 2,676.8 ha (Table 2).

### Sampling design and data collection

Within each delineated burn unit, a stratified random sampling approach was employed to establish vegetation monitoring plots. Plot centers were systematically located on the vertices of a grid, with stratification based on vegetation type and previous treatment (including thinning, pile burning, and previous fires) when applicable, and a 50-meter buffer zone was incorporated to mitigate edge effects and proximity to roads. We established 0.04 ha (11.4 m radius) permanent circular plots across burn units. All field data were collected using a modified version of the USDA Forest Service Common Stand Exam (CSE)^13^. This protocol includes quantification of overstory and understory structure, standing and downed trees, surface fuels and woody debris, and ground cover.

Field data were collected at each plot during multiple sampling periods. Pre-fire data were gathered 1 to 6 months before the prescribed fires, while immediate post-fire data were collected 1 to 4 weeks afterward. Follow-up measurements took place 1 year later, with additional sampling conducted at some sites at 2, 4, 5, and 8 years after the fires. Photo points were established at each end of the transect line, oriented at four azimuths, and photos were taken from each end towards the plot center during each sampling episode.

#### Tree measurements

Tree species, status (live or dead), DBH (cm), total height (m), and height to live crown (m) were recorded for all trees (≥1.37 m tall and DBH ≥7.62 cm), before and after prescribed fire treatments. Post-fire effects on trees were recorded immediately after prescribed fires and included survival, scorch, and torch heights (m) and percentages, and bole char height (m).

#### Ground and surface fuel load

Surface and ground fuels were sampled along four linear transects arrayed in each cardinal direction (N, S, E, W), starting at the plot edge and ending at plot center, using the Brown’s Transect method^14,15^. 1-hr (0–0.64 cm) and 10-hr (0.64–2.54 cm) fuels were tallied along the first 2 m of the transect line; 100-hr (2.54–7.62 cm) fuels along the first 4 m; and 1000-hr + ( >7.62 cm; aka coarse woody debris, CWD) fuels were tallied along the entire length of each transect. Duff and litter depth (cm) were measured at 2 points along each transect (at 0 m and 4 m from the transect edge). For CWD, the diameter at the point of intersection, length (m), and decay class were recorded individually. Fuel loads (t ha^-1^) were estimated using the Rfuels package^16^, based on Brown’s transects in forests dominated by Sierra Nevada conifer species.

#### Vegetation and ground measurements

Ocular estimates of percentage cover by trees, shrubs, grasses, and herbs were made in the entire 0.04 ha plot to the nearest 1%. Modal heights were recorded. Ground cover percentage data were visually estimated for bare soil, litter, rock (particles > 2 mm diameter), woody debris, basal vegetation, and burn piles (if present), with additional categories for post-fire sampling of ash substrate and black litter.

#### Species composition

All species were identified at the 0.04 ha plot, and percent cover and status (live/dead) were recorded for each species. If a species could not be confidently identified, genus was recorded instead. For trees, vegetation layer was noted (overstory, seedling, sapling, resprout) and for shrubs modal height (m) was recorded.

#### Tree regeneration

A smaller 0.006 ha circular plot, sampled at the CSE plot center, was used to record the number, species, and status of all saplings (≥1.37 m tall and DBH < 7.62 cm) and all seedlings (<1.37 m tall). For each sapling, diameter at breast height (DBH, cm) and height (m) was recorded. Only the height (cm) of the tallest seedling by species was recorded. For all resprouts, the number of sprouts originating from each resprout clump was recorded, as well as the height (m) of the tallest sprout.

## Data Records

**Fig. 3:**
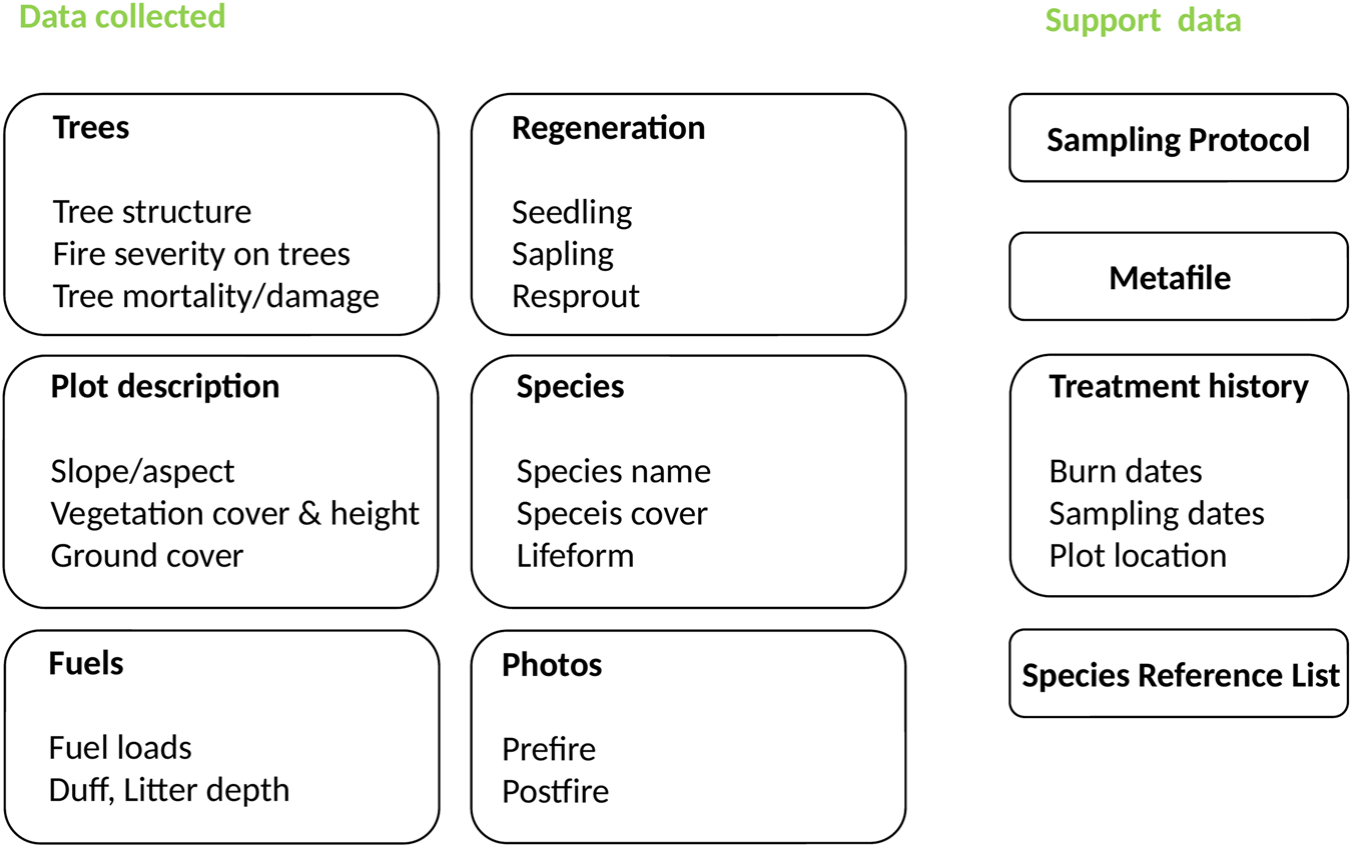
Dataset structure and supporting documentation. The dataset encompasses six primary data categories (left): tree characteristics including structure and fire-induced damage; plot-level descriptors such as topographic features and vegetation parameters; fuel load measurements including surface and ground fuel loads; regeneration dynamics through seedling, sapling, and resprout monitoring; species composition and abundance; and photos of pre-fire and post-fire for each plot. Supporting data elements (right) include standardized sampling protocol; metadata file; treatment history records documenting burn and sampling chronology with spatial coordinates; taxonomic reference materials. This integrated framework enables comprehensive assessment of fire effects and post-fire recovery trajectories across multiple temporal and spatial scales.

The CPFMP dataset is available at Dryad^17^ and consists of seven data tables provided as comma-separated text files (*.csv), two support documents provided in portable document format (*.pdf), and one folder (*.zip) containing all labeled photos as a joint photographic experts group format (*.jpg). Five of the tables include the main collected and calculated data (trees.csv, fuels.csv, plotd.csv, species.csv, regen.csv), and two additional data tables provide support data (bu_treatment.csv, species_referencelist.csv) (Fig. 3). The database comprises 36 sites, 114 burn units, 972 plots, and 1,838 total surveys conducted.

The trees.csv file contains individual tree measurements, including status (live or dead), species, DBH (cm), height (m), height to live crown (m), fire severity metrics (scorch height (m), torch height (m), scorch percent, torch percent), and notes of any tree damage, such as broken top, disease, pests. The fuels.csv file includes surface and ground fuel loading calculations by time lag classes (1-hr to 1000-hr + fuels), as well as duff and litter depths and loadings. The plotd.csv file provides descriptive information about each plot for each sampling time, such as slope, aspect, and vegetation and ground cover percentage measurements. The species.csv file details the vegetation composition of each plot, which includes the cover percentage of each identified plant species within the plots, the layer code of tree species, and the modal height of shrub species. The regen.csv file focuses on tree regeneration within the microplot, recording data on seedlings, saplings, and resprouts. The bu_treatment.csv file serves as a plot treatment and sampling log that includes IDs of the site, burn unit, prescribed burn dates, sampling types and dates, and geographic coordinates. The species_referencelist.csv file contains a complete list of all species codes recorded in all plots, along with their respective scientific names, lifeforms, and nativity status in California.

The first support document, CPFMP_MetaData.pdf, serves as a metadata file for the CPFMP field data collection operations and dataset. It includes descriptions of the tables within the datasets, explanations of special cases, and details of calculations performed on the data. This document contains essential information for data interpretation, including precise definitions for each variable, corresponding units of measurement, and a description of quality flags used to denote data provenance or limitations.

The second support document, CPFMP_Protocol.pdf, details the protocol that was followed during field data collection and was adapted from the CSE Field Guide^13^. It outlines the procedures for the sampling design, plot establishment, measurements of tree characteristics, vegetation cover and composition, ground cover, fuel loads, and regeneration.

Missing data points across all main tables were consistently represented by the code NA. Explanatory notes clarifying the reasons for missing data were included when available in CPFMP_MetaData.pdf. The data contained in this data descriptor have been deposited in Dryad^17^ and the final files have been carefully screened for errors.

Field data from the Caples site from prior sampling episodes (2013, 2017, and 2018) was incorporated into CPFMP dataset. These pre-fire data were standardized and integrated with subsequent monitoring data.

## Technical Validation

### Validation procedures

Multiple validation procedures were implemented to ensure data quality and reliability. Field crews underwent rigorous training before data collection.

Automated data validation scripts identified potential errors, including values outside biologically reasonable ranges, temporal inconsistencies (e.g., tree diameters decreasing over time), incongruences (e.g., scorch percentage smaller than torch percentage), and missing required data fields. Flagged entries underwent manual review, with original datasheets and field notes consulted to resolve discrepancies. When errors could not be resolved, data was corrected based on contextual evidence or discarded.

## Usage Notes

This dataset supports diverse research and management applications related to prescribed fire effects and ecosystem response. Potential applications include:

1. Fire Effects Modeling: Data can calibrate and validate fire effects models, particularly for California ecosystem types where empirical data have been limited.
2. Treatment Efficacy Assessment: Quantitative evidence of prescribed fire effectiveness for various management objectives (fuel reduction, ecological restoration, etc.).
3. Recovery Trajectory Analysis: Time-series data enable analysis of ecosystem recovery patterns across diverse environmental conditions.
4. Climate Change Implications: Dataset spans significant climate variability, allowing exploration of weather and climate influences on fire effects.
  When using these data, users should consider:
  Site Representation: While encompassing broad ecological diversity, the dataset overrepresents accessible areas and underrepresents extremely remote locations.
  Treatment Variability: Prescribed burns were conducted by different agencies with varying objectives and techniques, creating inherent variability in treatment implementation.
  Sampling Intensity: Sampling intensity varied between sites based on size and heterogeneity. Site-specific sample size should be considered when making cross-site comparisons.

### Future work

These data represent an ongoing program of work with continued funding and program support at CAL FIRE. While this dataset represents data collected and managed by UC Davis between 2019–2024 in partnership with CAL FIRE, CAL FIRE’s CPFMP remains active and will continue to add new data and project sites. CFPMP is continuing to develop Department-Academia agreements to continue the program and expand methods and analyses for other unique vegetation and fuel systems. Interested parties can contact CAL FIRE for status on new versions of the data set.

## Data Availability

The California Prescribed Fire Monitoring Program dataset 2019–2024 is openly available at Dryad (10.5061/dryad.612jm64gw)^17^.

## Code availability

No custom code was generated. The six main data tables can be directly downloaded from Dryad^17^ and imported into any spreadsheet, database or statistical software. For instance, in R, the files can be simply uploaded as follows: trees < - read.csv(“trees.csv”), fuels < - read.csv(“fuels.csv”), plotid < - read.csv(“plotid.csv”), species < - read.csv(“species.csv”), regen < - read.csv(“regen.csv”), bu_treatment < - read.csv(“bu_treatment.csv”).
